# Editorial: Asymmetry in the central nervous system: Functional implications

**DOI:** 10.3389/fnsys.2022.1002965

**Published:** 2022-09-07

**Authors:** Hugo Leite-Almeida, Paola d'Ascanio, Ugo Faraguna, Diego Manzoni

**Affiliations:** ^1^School of Medicine, Life and Health Sciences Research Institute (ICVS), University of Minho, Braga, Portugal; ^2^ICVS/3B's, PT Government Associate Laboratory, Braga/Guimarães, Portugal; ^3^Clinical Academic Center—Braga, Braga, Portugal; ^4^Department of Translational Research and of New Surgical and Medical Technologies, University of Pisa, Pisa, Italy; ^5^Department of Developmental Neuroscience, IRCCS Fondazione Stella Maris, Pisa, Italy

**Keywords:** CNS (a)symmetry, brain hemispheres, cortex, callosal connections, lateralizarion

The functional processing of several brain functions is asymmetrically distributed between homotopic hemispheric areas, so that lateralized cortical lesions may result in very specific functional loss (Esteves et al., 2019, 2020, 2021). Paradoxically, the cognitive effects of unilateral lesions can be rescued by a second contralateral one, as elegantly demonstrated by Lomber and Payne (1996). Beyond this extreme experimental evidence, recent experiments suggest that asymmetry in the trigeminal sensory inputs related to occlusal factors may lead to deficits in cognitive functions by affecting the balance of the brain ascending modulatory systems. The goal of this Research Topic was to focus on the neurophysiological and molecular mechanisms underlying symmetry maintenance and/or disruption, as well as their outcome in behavioral and adaptive terms.

In the present topic two papers have studied the impact that asymmetries in the sensorimotor trigeminal activity might have on the brain. Tramonti Fantozzi et al. demonstrated that the presence of an asymmetry in the development of occlusal forces gates the stimulating effects of unilateral chewing on performance and arousal, so that improvements occur only when mastication takes place on the side displaying the lower EMG activity of elevator muscles during clenching. On a related topic, Viggiano et al. showed how unilateral chewing activity increased the local blood flow at the level of the ipsilateral trigeminal complex only when this activity was performed on the right side. These studies emphasize that asymmetries in trigeminal activity and local perfusion may underline a loss in cognitive performance and prime the development of migraine attacks.

Regarding the vestibular system, asymmetrical activity may impact on the cognitive sphere, as indicated by the paper of Borel et al. addressing spatial orientation alterations following unilateral neurotomy. Intriguingly, these experiments demonstrate that the chronic deficits induced by lesions on either side are consistent with a decreased activity of the right parietal region. Thus, the notion according to which asymmetries in the afferent input systems may alter the hemispheric balance leading to functional impairment found some support in these studies. According to Tramonti Fantozzi et al. such hemispheric imbalance may arise from the action that sensory systems exert on the structures of Ascending Reticular Activating Systems (such as the Locus Coeruleus), which control hemispheric excitability. Sensory asymmetries, however do not necessarily act on symmetric, balanced brain networks.

Mundorf et al. analyzed structural asymmetries across different neurodevelopmental and psychiatric disorders from large scale neuroimaging studies. Almost all disorders were associated with alterations in structural hemispheric asymmetries. Surprisingly, major depressive disorder was an exception to this pattern which will certainly be object of further studies in the future. In a similar vein, the paper by Deemyad addresses more specifically the asymmetry in somatosensory and auditory cortical structures associated with language processing in Autistic Spectrum Disorders, discussing the importance of cortical dominance and interhemispheric disruption of balance between excitatory and inhibitory synapses as pathophysiological mechanisms of this pathological condition. Finally, beyond the asymmetries in the sensory afferent input which may alter the balance and/or the distribution of specific functions across hemisphere, also deficits of the intra-hemispheric (callosal) connections may lead to impairment of specific functions, as shown by Fabri et al. for the imitative behavior.

Overall these six papers point to the principle that changing the normal balance of activity or the normal distribution of neural processing between the hemisphere may lead to loss of specific functions. The last two papers link the cognitive to the posture-kinetic domain, showing that lesion-induced neural asymmetries may lead to a disruption of the ontogenetic establishment of a symmetric body (Gordy and Straka), but also elicits reactions at cellular, synaptic or network level that tend to restore the lost functional symmetry in the sensory-motor domains of the Central Nervous System (Le Ray and Guayasamin).

In conclusion, the “*Asymmetry in the Central Nervous System: Functional Implications*” papers support the view that both motor and cognitive domains are shaped by the nervous system asymmetric structure and function. While not entirely clear the adaptive gain or loss of such asymmetries, this knowledge has already prompted the use of sensory rebalancing techniques within the neuro-rehabilitation field.

## Author contributions

DM wrote the initial draft. All authors contributed to the final version.

## Conflict of interest

The authors declare that the research was conducted in the absence of any commercial or financial relationships that could be construed as a potential conflict of interest.

## Publisher's note

All claims expressed in this article are solely those of the authors and do not necessarily represent those of their affiliated organizations, or those of the publisher, the editors and the reviewers. Any product that may be evaluated in this article, or claim that may be made by its manufacturer, is not guaranteed or endorsed by the publisher.
